# Facilitatory Effects of Multi-Word Units in Lexical Processing and Word Learning: A Computational Investigation

**DOI:** 10.3389/fpsyg.2017.00555

**Published:** 2017-04-13

**Authors:** Robert Grimm, Giovanni Cassani, Steven Gillis, Walter Daelemans

**Affiliations:** Department of Linguistics, Computational Linguistics and Psycholinguistics Research Center, University of AntwerpAntwerp, Belgium

**Keywords:** multi-word units, age of first production, reaction times, contextual diversity, language acquisition, word learning, lexical processing

## Abstract

Previous studies have suggested that children and adults form cognitive representations of co-occurring word sequences. We propose (1) that the formation of such multi-word unit (MWU) representations precedes and facilitates the formation of single-word representations in children and thus benefits word learning, and (2) that MWU representations facilitate adult word recognition and thus benefit lexical processing. Using a modified version of an existing computational model (McCauley and Christiansen, 2014), we extract MWUs from a corpus of child-directed speech (CDS) and a corpus of conversations among adults. We then correlate the number of MWUs within which each word appears with (1) age of first production and (2) adult reaction times on a word recognition task. In doing so, we take care to control for the effect of word frequency, as frequent words will naturally tend to occur in many MWUs. We also compare results to a baseline model which randomly groups words into sequences—and find that MWUs have a unique facilitatory effect on both response variables, suggesting that they benefit word learning in children and word recognition in adults. The effect is strongest on age of first production, implying that MWUs are comparatively more important for word learning than for adult lexical processing. We discuss possible underlying mechanisms and formulate testable predictions.

## 1. Introduction

In this paper, we examine the role of lexicalized word combinations in (1) child word learning and (2) adult lexical processing. Consider, for example, sequences whose meanings cannot be derived from the meaning of their constituent words (e.g., *leave of absence, high five*, or *kick the bucket*). Due to their semantic opacity, such expressions are likely to be stored wholesale in long-term memory. But even non-compositional sequences such as *don't have to worry* or *I want to go* appear to be represented as units in their own right (Arnon and Snider, 2010). Here, we use the term *multi-word unit* (MWU) to refer to any sequence of words—semantically opaque or not—which is likely to be lexicalized; and by using a modified version of an existing computational model (McCauley and Christiansen, 2014) which forms MWUs by relying on transitional probabilities between words, we operationalize MWUs as particularly internally cohesive word sequences.

To date, MWUs have been investigated in studies on first language acquisition (Bannard and Matthews, 2008; Arnon and Clark, 2011; McCauley and Christiansen, 2014) as well as in work concerned with adult processing (Arnon and Snider, 2010; Arnon and Priva, 2014). Findings in the two areas suggest that both adults and children possess cognitive representations of MWUs. In addition, Arnon and Clark (2011) provide experimental evidence that MWUs facilitate the acquisition of smaller linguistic units contained within them. Together, the available evidence suggests a developmental pattern from MWU to single-word representations, with a beneficial effect of the former on the acquisition of the latter. Based on this, we hypothesize that children sometimes form MWU representations before they form representations of the words contained within them, and that these MWU representations then facilitate the acquisition of single-word representations. We dub this the *MWU acquisition hypothesis*.

We furthermore propose that MWU representations interact not just with the acquisition of individual words in children but also with the processing of individual words in adult cognition. This proposal is motivated by a strand of research concerned with the contextual distribution of words (McDonald and Shillcock, 2001; Adelman et al., 2006; Johns et al., 2012, 2014; Jones et al., 2012). Generally, increased contextual diversity (measured in terms of documents or co-occurring context words) is associated with faster word recognition in adults. We suggest a link between findings relating to MWUs and to contextual diversity: high contextual diversity of words will lead to the formation MWU representations containing such words. Therefore, just like contextual diversity, MWUs are expected to be associated with faster lexical processing in adults. Thus, we hypothesize that MWU representations facilitate recognition of the individual words contained within them—a proposal which we refer to as the *MWU processing hypothesis*. In the following, we describe in more detail the findings on which we base the two hypotheses as well as how we evaluate them in this study.

We turn first toward the language acquisition literature. Here, MWUs have emerged as a key theoretical concept in usage-based approaches (Behrens, 2009; Tomasello, 2009). Within this broad theoretical framework, learners' linguistic representations are conceived of as continually complexifying entities, with the developed cognitive system containing both lexically specific and more abstract patterns. At early stages in development, most representations are lexically specific, and child language is “(partially) formulaic and item-based” (Behrens, 2009, p. 393). In other words, child language development is thought to involve representations which are lexically specific and span multiple words.

Observations to this effect have been made by several researchers. Peters's (1983) surveyed various examples, concluding that many of the early linguistic units acquired by children consist of more than one word and are often not yet analyzed in terms of their constituent parts. For example, Clark (1974) reported child utterances such as *I don't know where's Emma one*, which appear to consist of two previously heard utterances (*I don't know* and *where's Emma one*)—the implication being that the child must have treated each of these utterances as a single unit. Similarly, Tomasello (1992) reported that his daughter first began using the verb *find* as part of the utterance *find-it* during her 17th month, apparently to express a desire for an absent object (e.g., a particular toy). It was only at later stages that she started to generalize usage: first, she began to use *find-it* in combination with particular object names—as in *find-it bird*; and finally, at 20–24 months, she began to use *find* together with function words like pronouns and articles.

Tomasello (2000) reviews studies which suggest that a gradual development from lexically specific to more general language use is the norm. In a frequently used paradigm, young children are taught novel verbs—e.g., *tam*, as in *Jim is tamming*. Later, they are given the opportunity to use the verb in novel syntactic constructions, such as the transitive sentence *Jim is tamming the car*. Aggregating findings from several such studies shows that the proportion of children who generalize usage of novel verbs from intransitive to transitive sentences increases with age, with around 10% of children generalizing at 2 years and close to 100% generalizing at 8 years (cf. Tomasello, 2000, p. 223).

There is thus evidence that children's early utterances are lexically specific, whereas adults appear to be more easily capable of productive language use. This in turn suggests that some early representations are *fossilized MWUs*: representations which span multiple words, with usage restricted to particular situations and to particular communicative purposes. It is only at later stages in development that children begin to form single-word representations, which then leads to more productive language use.

Experimental evidence for the existence of children's MWU representations is provided by Bannard and Matthews (2008), who presented 2 and 3 year-olds with frequent MWUs like *a drink of tea* and matched infrequent MWUs like *a drink of milk* that differed in the last word. Two and three year-olds were faster to repeat frequent MWUs, and 3 year-olds were also faster to repeat the first three words if they formed a frequent MWU with the fourth word. Since the final word and the final bigram (e.g., *of tea* and *of milk*) were matched for frequency, the processing advantage for frequent MWUs can only be attributed to the frequency of the entire MWU, rather than to the frequencies of its component words, suggesting that children have access to cognitive representations of MWUs. Bannard and Matthews (2008) argue, furthermore, that their subjects were likely familiar with the words contained in the MWUs, which implies the co-existence of MWU and single-word representations. The same argument can be made for adults, who are faster to recognize and produce frequent four-word MWUs in similar experiments (Arnon and Snider, 2010; Arnon and Priva, 2014).

One of the emerging patterns in language acquisition, then, is that children's early lexical representations span multiple words. In addition, Arnon and Clark (2011) found that MWUs interact with the acquisition of morphemes: in their study, 4–6 year-olds produced more correct irregular plurals after familiar lexically specific frames than after general questions. Subjects were presented with depictions of several object instances. The object name was elicited either with a labeling question or with a lexically specific frame. For example, on one particular trial the objects were sheep, the lexically specific frame was *Count some–*, and the labeling question was *What are all these called?* 4–6 year-olds were more likely to complete the lexically specific frame with *sheep* and would provide relatively more incorrect plural forms—like the over-regularized *sheeps*—in response to the labeling question. This suggests that MWUs like *count some sheep* affect the way in which some of the smaller units contained within them are learned.

Given the evidence, it seems natural to suggest not only that children's early lexical representations often span several words, but also that such MWU representations facilitate the language acquisition process (cf. Arnon, 2009). In particular, we propose the *MWU acquisition hypothesis*, according to which the formation of MWU representations precedes and facilitates the formation of single-word representations. Since adults, like children, appear to possess MWU representations (Arnon and Snider, 2010; Arnon and Priva, 2014), we suggest that MWUs also facilitate the processing of individual words in adult cognition (*MWU processing hypothesis*). We do not have experimental evidence indicative of a facilitatory effect of MWUs on adult lexical processing, but we can nevertheless derive indirect evidence from a strand of research concerned with the effect of contextual diversity on word recognition.

Several studies have investigated the effect of contextual diversity (henceforth CD) on adult lexical processing. In a corpus-based analysis, Adelman et al. (2006) counted the number of documents in which each target word occurred and found the resultant measure of CD to be more predictive of reaction times in word naming and lexical decision tasks than raw frequency counts. Their approach has since been refined by Jones et al. (2012), who weighted document counts relative to semantic overlap among documents and achieved an even better fit. Since both studies relied on naming and lexical decision data collected via *visual* word naming and recognition tasks, it is possible that the results are an artifact of modality. Johns et al. (2012) addressed this caveat by using data from an auditory word recognition task and found similar effects of CD.

Experimental evidence for a facilitatory effect of CD was collected by Johns et al. (2014). Adult subjects were presented with reading passages, each containing a low-frequency word which was replaced by a novel word form (the target). In a low-CD condition, targets were embedded in reading passages taken from a single discourse topic. In a corresponding high-CD condition, targets appeared across passages from different topics. After the reading phase, subjects performed a pseudo-lexical decision task, wherein targets presented in the high-CD condition were recognized faster and more accurately.

There is thus evidence that CD, defined on a paragraph or document level, increases the speed with which adults recognize written and spoken word forms. This is mirrored by the effect of more locally defined contextual diversity. McDonald and Shillcock (2001) counted co-occurring context words, within a small window to the left and right of each target word, and measured the divergence (relative entropy) between each target's context word distribution and a baseline frequency distribution. Target words where this divergence is large tend to be associated with longer lexical decision latencies, which suggests that words which appear in relatively specific local contexts are harder to recognize. Put differently, words whose context of use is relatively limited are hard to recognize, whereas words that can be used together with a broad range of context words are easy to recognize. This implies, to borrow McDonald and Shillcock (2001)'s phrasing, that “exposure to the context in which a given word is spoken contributes to aspects of that context being encoded in the word's mental representation” (p. 301). In the present study, we would say that co-occurrence with context words—or high CD—leads to the formation of MWU representations.

Further evidence that such a process could unfold in the human mind was collected by Hills et al. (2010). Their study takes as a starting point the previous observation that age of acquisition and adult-generated free associates are negatively correlated (Hills et al., 2009). Associates are generated by presenting a cue word (e.g., *cat*) to adult subjects, who then give back the first word that comes to mind (the target, e.g., *mouse*). The number of different cues for which a target is provided (the *indegree* of the target) is negatively correlated with age of acquisition—i.e., words with a high indegree tend to be acquired at relatively early ages. Hills et al. (2010) show that indegree is positively correlated with the number of different context words that co-occur with the target in a corpus of child-directed speech (CDS)—presumably because children link words to one another if they co-occur in the input. That is, the latter correlation is probably responsible for the former: CD likely leads to the internal linking-together of co-occurring words, which appears to facilitate the acquisition of individual words.

We can directly connect this result to Arnon and Clark (2011)'s study: Arnon and Clark (2011) found that MWUs affect the acquisition of irregular plural morphemes, while Hills et al. (2010)'s results suggest that linking words to one another—i.e., the formation of MWU representations—is likely to affect the acquisition of individual words. The formation of MWU representations, in other words, appears to affect the acquisition of smaller linguistic units (e.g., words or morphemes) contained within them. It is reasonable, then, to expect an effect of MWUs not just on word learning in children but *also* on adult word recognition. After all, a range of studies have demonstrated an effect of CD on the speed with which adults recognize words; hence, if CD leads to the formation of MWU representations, we should expect MWU representations to facilitate word recognition in adults.

Based on the reviewed findings, we have proposed two hypotheses: according to the *MWU acquisition hypothesis*, the formation of MWU representations precedes and facilitates the formation of single-word representations in children; and according to the *MWU processing hypothesis*, MWU representations facilitate the processing of individual words in adults. In this study, our primary objective is the evaluation of the two hypotheses via correlational analysis.

Concretely, we use an existing computational model (McCauley and Christiansen, 2011, 2014), with minor modifications designed to make the output more noise-resistant, to extract MWUs from a corpus. The kinds of MWUs the model discovers have previously been used (cf. McCauley and Christiansen, 2014) to match results from Arnon and Clark (2011) and Bannard and Matthews (2008), which gives credence to their suitability as approximations of the types of MWUs human learners might discover. After running the model on two different corpora, we use the number of MWUs within which a given word is contained as an independent variable. If the *MWU acquisition hypothesis* is true, words contained in many different MWUs should be easier to acquire than words contained in fewer MWUs. Likewise, if the *MWU processing hypothesis* is true, such words should also be easier to process. To see why, suppose that the model discovers a large number of different MWUs which each contain a particular target word *X*. We do not know if human learners, given similar input, would discover the exact same MWUs; but our expectation is that the more MWUs containing *X* are discovered by the model, the more likely human learners would be to form cognitive representations of MWUs that also contain *X*. And if the *MWU acquisition hypothesis* is true, the formation of such MWU representations should facilitate the acquisition of words contained within them. Thus, *X* should be easier to acquire than words which appear in fewer MWUs. Similarly, if the *MWU processing hypothesis* is true, representations of MWUs containing *X* should facilitate processing of *X*—and hence, *X* should be easier to process than words contained in fewer MWUs.

To track word learning in children and lexical processing in adults, we use two response variables: age of first production (AoFP) and adult reaction times (RTs) from a lexical decision task. AoFP serves as an index of word learning: if a word is first produced relatively early in development, we assume that this is in part because it is easy to learn when and how to use it. Likewise, if first production occurs comparatively late, we assume that this reflects difficulties in establishing when and how to use the word. Next to AoFP, we use RTs from a lexical decision task to measure word recognition in adults: words with fast RTs are easier to recognize, relative to words with slow RTs. Correlating the number of different MWUs per target word with AoFP and adult RTs thus allows us to measure (a) the potential impact of MWUs on child word learning and (b) their potential impact on adult word recognition. In line with our two hypotheses, we expect that words contained in many MWUs will be first produced at relatively early stages in development and will be recognized relatively quickly in a lexical decision task. In other words, we expect the independent variable to correlate negatively with both RTs and AoFP.

Our first and primary goal is to test this prediction via correlational analysis. In doing so, we attempt to control for the frequency of target words, since frequent target words will also tend to appear within many MWUs. Beyond that, we aim to compare the effect of MWUs across word recognition and word learning—i.e., we ask which of the two areas is potentially more strongly affected by MWUs. Here, we have no a priori reason to expect a stronger effect on one over the other area: given that children's early utterances are lexically specific MWUs, it could be that language acquisition interacts particularly strongly with MWUs; but it is also possible that MWU representations become more entrenched over the course of development and thus become even more central to adult processing.

## 2. Analysis I: extracting multi-word units from child- and adult-directed speech

In this first analysis, we use a modified version of an existing computational model (McCauley and Christiansen, 2011, 2014) to extract MWUs from a corpus of transcribed CDS and a size-matched corpus of transcribed informal conversations among adults. The types of MWUs the model discovers—sequences with particularly strong transitional probabilities between constituent words—have previously been used to model results with respect to the role of MWUs in child language acquisition (McCauley and Christiansen, 2014), providing empirical support for their cognitive relevance. By running the model on a corpus of CDS, we aim to approximate the types of MWU representations that children would discover; and by running the model on a corpus of transcribed speech exchanged among adults, we aim to approximate the types of MWU representations that adults might possess. The two sets of MWUs then serve as the basis for calculating the independent variable used in the subsequent correlational analyses: the number of MWUs per target word.

### 2.1. Method

#### 2.1.1. Model

The computational mechanism we use to discover MWUs is a modified version of a model developed by McCauley and Christiansen (2011, 2014). In a first phase, their model—called Chunk-Based Learner (CBL)—extracts MWUs from a corpus of CDS. In a second phase, it generates child-produced utterances based on discovered MWUs. The full model is described in McCauley and Christiansen (2011, 2014), along with how it can be used to generate child productions and model results from Bannard and Matthews (2008) and Arnon and Clark (2011). Here, we provide a brief description of the component responsible for the discovery of MWUs, as well as how we modified it in order to reduce the impact of noisy input.

The CBL is psychologically motivated in that (1) it processes a given corpus in an incremental fashion— i.e., utterance by utterance and word by word—, and (2) it relies on backward transitional probabilities (BTPs), which human learners are sensitive to (Pelucchi et al., 2009). In addition, it does not require parameters governing MWU length or frequency. For example, consider the selection of common word sequences as a possible way of extracting MWUs from a corpus. With such a method, we would have to define both a maximum MWU length as well as an arbitrary frequency threshold for a word sequence to count as an MWU. The CBL, in contrast, utilizes BTPs between words as the only criterion for inclusion into MWUs. We can conceptualize the model as a psychologically grounded method for segmenting a corpus into MWUs which are, by virtue of the BTPs between component words, more internally cohesive than randomly selected word sequences.

More formally, processing an utterance *u* is initiated by incrementing the frequency count of the first word *w*_1_ by 1 and creating a new MWU with *w*_1_ as its only member. For each subsequent word *w*_*i*_ at utterance position 1 < *i* ≤ *length*(*u*), the model keeps track of the number of times *w*_*i*_ has been encountered so far, as well as how often the immediately preceding word *w*_*i*−1_ has occurred one position to the left of *w*. The model then calculates the BTP of *w*_*i*_ and *w*_*i*−1_: *p*(*w*_*i*−1_|*w*_*i*_). If this conditional probability is larger than the average BTP, across all words which have occurred one position to the left of *w* in all utterances so far considered, *w*_*i*_ is added to the current MWU. Else, the current MWU is added to a set *M*, and a new MWU is created—again with *w*_*i*_ as its only member. Once the model has formed a first set of MWUs, it uses them as a resource to constrain the formation of future MWUs: if a sequence of words *w*_*i*−1_, *w*_*i*_ constitutes part of an existing MWU, future occurrences of *w*_*i*−1_ and *w*_*i*_ are grouped into an MWU regardless of the BTP between the two words. In this way, the model discovers MWUs of size 2 or larger, as well as single-word units, collected in *M*.

As mentioned, we introduce a minor modification to the CBL. In the original version, two given words form part of an MWU if the BTP between them is larger than average. However, for words which the model has not yet encountered very often, BTP may be quite noisy. This is a matter of sample size: statistics estimated from small samples can be strongly influenced by aberrations in the data, and BTPs calculated on the basis of very low frequency counts could be biased by a number of possible peculiarities (e.g., a particular topic of conversation, a non-standard dialect, transcription errors, and so on). As words are encountered more often, the effect of noise will diminish, and BTPs will become more representative of general language use. To guard against noise at early stages of learning, when BTPs may be unstable, we weigh the decision to group words into MWUs by the amount of prior experience: a given word *w*_*i*_ and the immediately preceding word *w*_*i*−1_ are included in an MWU only if the BTP between them is larger than the mean BTP *plus* the reciprocal of the frequency count of *w*_*i*_. That way, words can still be included in MWUs even if the model has had relatively little exposure to them, but only if the BTP with preceding words is comparatively large. As words are processed more often, this effect diminishes exponentially—in line with the increasing stability of BTPs.

We consider the MWUs discovered in this fashion as approximations of the types of MWU representations created by human learners—the underlying assumption being that internally cohesive sequences of words are good candidates for cognitively plausible MWUs. This assumption derives its justification from the fact that the MWUs discovered by the CBL can be used to model results from Bannard and Matthews (2008) and Arnon and Clark (2011)—cf. McCauley and Christiansen (2014)—two key studies which motivated our hypothesis regarding the effect of MWUs on word learning. This track record notwithstanding, there is of course no guarantee that a particular MWU discovered by the CBL is really represented in the minds of language users, but it is our expectation that model-derived MWUs are more likely to be cognitively represented than randomly selected word sequences. In the following analyses, we attempt to confirm this via comparison to a random baseline. The baseline model operates just like the CBL, except insofar as it randomly decides whether or not to group two successive words into an MWU. That is, the baseline model also incrementally processes a given input utterance, considering each word for inclusion into an MWU. But instead of using BTP to decide whether or not the current and the preceding word form part of an MWU, it relies on a random coin toss to make that decision. To avoid confusion, we refer to the units discovered by the baseline as *word sequences*, whereas we continue to use the term *MWUs* to refer to the units discovered by the CBL.

#### 2.1.2. Corpora

McCauley and Christiansen (2011, 2014) used a corpus of CDS to discover MWUs with the CBL. Children learn primarily in the context of CDS, which differs quite markedly from the type of speech used by adults to address other adults (adult-directed speech, henceforth ADS). Among other things, CDS consists of shorter phrases, contains more pauses, shows a wider range of pitches, and is composed of a limited vocabulary (Saxton, 2010). These differences are, in turn, likely to affect the language acquisition process at various levels (Matychuk, 2005; Saxton, 2009). It makes sense that McCauley and Christiansen (2011, 2014)—modeling child-produced speech and child-elicited experimental results—chose an input corpus that reflects the unique linguistic environment of English-speaking children.

In the current study, however, we are interested in adult processing *in addition to* language acquisition. If we were to use a corpus of CDS, we would implicitly claim that adult lexical processing and child word learning are equally strongly affected by MWUs found in CDS—even though adults' primary linguistic input differs substantially from CDS. We address this challenge by using two different input corpora: one that is similar to the collection of corpora used by McCauley and Christiansen (2011, 2014), and an additional size-matched corpus of ADS. When carrying out correlational analyses, we then assume that MWUs in CDS are a more direct determinant of word learning in children, whereas MWUs in ADS are a more direct determinant of adult lexical processing. Consequently, when measuring the effect of MWUs on child word learning, we base the analyses on MWUs found in CDS; and when examining the effect of MWUs on adult lexical processing, we focus on MWUs found in ADS.

The CDS corpus is based on eight British English corpora from the CHILDES database (MacWhinney, 2000) (cf. Appendix A in Supplementary Material for an enumeration). An aggregated CDS corpus is created by first ordering the transcripts from all included corpora by the age of the child addressed in each transcript. We then extract all utterances made by any adult whose utterances were transcribed (usually the mother or father of the child or children in question, sometimes another relative, or an experimental confederate). The full CDS corpus contains 4,869,472 tokens of CDS, produced by 201 adults in interactions with 133 different children.

By aggregating different corpora, we are conflating the language directed at children from different backgrounds. However, limiting ourselves to particular CHILDES corpora severely restricts the amount of available data, while working with data from several corpora is likely to increase the detectability of robust, corpus-independent patterns. At the same time, we include only British English corpora and exclude American English corpora, which increases comparability with the size-matched ADS corpus.

The ADS corpus is based on the informal spoken component of the 100-million-word British National Corpus (henceforth BNC) (Burnard, 2007), a resource designed to represent a wide cross-section of spoken and written British English. Due to the methodological challenges inherent in collecting representative spoken samples, the BNC mostly consists of written material. The spoken component comprises 10.58 million tokens, 6.28 million of which cover rather formal spoken English. The remaining 4.30 million tokens consist of transcribed conversations among adults, collected from 124 adult respondents who were given a recording device, together with instructions to record their everyday conversations. Except for the respondents, interlocutors were not aware of being recorded. Transcribed material was then included in the corpus only if all interlocutors had given consent upon being informed of the recordings. This informal spoken component of the BNC is a suitable source of ADS to compare against the CDS corpus.

The CDS and ADS corpora are taken from the same variety of English (British English) and are similar with respect to the number of tokens and interlocutors. Important differences have to do with the number of word types and the mean utterance length (cf. Table 1). Despite containing a similar number of tokens each, the CDS corpus contains 30% fewer word types than the ADS corpus, with utterances in the ADS corpus being on average two tokens longer than utterances in the CDS corpus. These differences are expected and likely reflect general differences between ADS and CDS (Saxton, 2010).

**Table 1:** **Relevant statistics for the ADS and CDS corpora**.

**Measure**	**Adult-directed speech**	**Child-directed speech**
nr. adult speakers	124	201
nr. tokens	4,233,645	4,869,472
nr. types	34,267	25,109
Median utterance length	5 (IQR: 7)	4 (IQR: 4)

### 2.2. Results and discussion

Running the CBL on the ADS and CDS corpora results in two different sets of MWUs—Table 2 summarizes relevant statistics about their distribution (upper section). There are relatively fewer MWU tokens (first row) and relatively more MWU types (second row) in ADS, while the median number of tokens per MWU (third row) is a bit smaller in CDS. And even though the overall statistics are roughly similar, the baseline model extracts comparatively more unique word sequences from CDS, with the median length of sequences from both ADS and CDS being larger than the corresponding lengths of CBL-extracted MWUs. This indicates that the MWUs discovered by the CBL deviate from randomly selected word sequences.

**Table 2:** **Relevant statistics about the distribution of MWUs in ADS and CDS**.

**Model**	**Measure**	**ADS**	**CDS**
Chunk-Based Learner	nr. MWU tokens	834,205	1,117,465
	nr. MWU types	495,610	467,849
	Median MWU length	5 (IQR: 4)	4 (IQR: 3)
Random baseline	nr. word sequence tokens	663,953	955,698
	nr. word sequence types	520,482	592,735
	Median word sequence length	6 (IQR: 4)	5 (IQR: 3)

*Upper section: MWUs extracted by the Chunk-Based Learner. Lower section: MWUs extracted by the random baseline model*.

The MWUs discovered by the CBL have a tendency to span comparatively more tokens as they decrease in frequency. For example, the five most frequent MWUs in the CDS corpus are *that's right* (frequency count: 5,705), *oh dear* (4,566), *is it* (4,538), *isn't it* (4,445), and *come on* (4,410). The five most frequent MWUs in the ADS corpus are *you know* (2,644), *oh yeah* (2,028), *is it* (1,797), *it is* (1,754), and *isn't it* (1,650). Among the lower-frequency MWUs, we find constructions such as *knife and fork* (CDS, with a frequency of 7), *glass of wine* (CDS, 4), *come across* (CDS, 3), *point of view* (ADS, 12), *I apologize* (ADS, 2), or *beg pardon* (ADS, 2).

The most frequent word sequences extracted by the baseline model often overlap with the most frequent MWUs discovered by the CBL: the baseline is bound to extract many of the short and frequent MWUs which the CBL discovers, simply because they are so frequent that even a random method will discover them by chance. As we consider less and less frequent MWUs, however, the degree of overlap weakens. For example, the overlap between the top 5,000 CBL-derived MWUs and the top 5,000 baseline-derived word sequences is 70% for ADS and 66% for CDS, but this shrinks to 36 and 49% if we consider the top 100,000 units. There is thus a principled difference in the types of MWUs discovered by the CBL and the word sequences extracted by the random baseline, in spite of the considerable overlap between the most frequent items. In the subsequent analyses, this should be reflected in a difference between results obtained with the CBL-extracted MWUs and results obtained with the baseline word sequences.

To derive the key independent variable for the remaining analyses, we count the number of different MWUs within which each target word appears. For example, suppose our target words are *boy, sit*, and *nice*. We would then consult the two sets of MWUs and count, for each of the three words, all MWUs which contain the word. We find that *boy* appears within 1,725 different CDS MWUs and 510 different ADS MWUs, *sit* within 3,046 CDS MWUs and 1,122 ADS MWUs, and *nice* within 3,838 CDS MWUs and 2,527 ADS MWUs. To illustrate the types of MWUs we have counted, Table 3 lists two high- and two low-frequency MWUs, in CDS and ADS, for each of the three example words.

**Table 3:** **The two most frequent and two of the least frequent MWUs for the three target words ***boy, sit***, and ***nice*****.

**Target word**	**ADS MWU**	**CDS MWU**	**ADS freq**.	**CDS freq**.
Boy	Good boy	Good boy	116	736
	Old boy	Clever boy	35	301
	There is a clever boy	Poor little boy	3	3
	Good old boy	Oh you naughty boy	2	2
Sit	Sit down	Sit down	106	324
	Sit there	Sit up	19	107
	Sit in the back	Sit on your chair	3	3
	You sit there	Can I sit down	2	2
Nice	Very nice	That's nice	132	354
	That is nice	Is that nice	88	219
	Isn't it nice	Do I look nice	3	3
	You look nice	Looks quite nice	2	2

*The right-most two columns contain MWU frequencies*.

In the immediately following analysis, we (1) evaluate the impact of this variable on child word learning and adult word recognition and (2) verify the assumption that ADS is the relevant linguistic input for adults, while CDS is the relevant input for children. Following this, in analysis III, we compare the results from analysis II to results obtained with the baseline model. Lastly, in analysis IV, we compare the effect of MWUs on word learning to their effect on word recognition.

## 3. Analysis II: evaluating the effect of multi-word units on word learning and word recognition

We now turn to the first of three correlational analyses. Here, our primary objective is to evaluate the impact of MWUs on word learning in children as well as on word recognition in adults. In line with the *MWU acquisition hypothesis*, we expect a beneficial effect of MWUs on the former; and given the *MWU processing hypothesis*, we also expect a facilitatory effect on the latter.

We use corpus-derived age of first production (AoFP) estimates to track word learning and reaction times (RTs) from a lexical decision task to track word recognition. Given a set of words with associated RT and AoFP values (henceforth *target words*), it is easy to count the number of MWUs within which each target word appears (a measure denoted by *#MWUs*). In addition, we also count how often individual target words occur within each corpus (denoted by *#Freq*). These predictors are then correlated with RTs and AoFP. We perform both full as well as partial correlations—correlating #Freq with the dependent variables while controlling for #MWUs, and correlating #MWUs with the dependent variables while controlling for #Freq. In each case, we expect the correlation coefficient to be negative: more frequent words should be recognized more quickly and learned earlier than less frequent words, and similarly for words appearing within a large number of different MWUs.

Recall that we use MWUs estimated from ADS and CDS to account for differences in the input received by adults and children. We assume that the linguistic input received by adults is best approximated by the ADS corpus, whereas the linguistic input received by children is best approximated by the CDS corpus. Thus, the two independent variables (#MWUs and #Freq) are estimated from CDS and ADS, resulting in two variants each: ADS-#Freq and CDS-#Freq, as well as ADS-#MWUs, and CDS-#MWUs. Since RTs are elicited from adult subjects, we consider the ADS variants relevant for their analysis; and since AoFP is based on child productions, we consider the CDS variants relevant for the correlations with AoFP. Given these assumptions, it would be methodologically dubious to correlate the two CDS predictors with RTs, or the two ADS predictors with AoFP. Nevertheless, instead of ignoring these possible correlations, we compare the CDS predictors to their ADS counterparts. If our reasoning is correct, we should expect RTs to correlate more strongly with the ADS predictors, while AoFP should correlate more strongly with the CDS predictors.

### 3.1. Method

#### 3.1.1. Target words

The set of target words consists of all word forms which occur in both the CDS and the ADS corpus and for which both AoFP and RT estimates are available (7,481 words). Target words are based on raw word forms, without any kind of pre-processing (e.g., stemming, lemmatization, or part-of-speech tagging).

#### 3.1.2. Age of first production

The first of two dependent variables, AoFP, measures word learning in children. Our reasoning is that words which are first produced early in development are easier to learn than words which are produced later. Ease of learning is likely determined by various factors, such as frequency in the child's input. Thus, a negative correlation between e.g., CDS-#Freq and AoFP would indicate that early-learned words are frequent in CDS; and a plausible interpretation would be that frequency of exposure leads to early word learning by exerting a facilitatory effect on one or more of the various processes involved in word learning.

We estimate AoFP from the transcribed speech of children addressed in a second collection of CHILDES corpora, without overlap with the CDS corpus. The rationale for using a second collection of corpora has to do with a possible confound. In the current study, we evaluate the effect of MWUs in ADS and CDS on two dependent variables—AoFP and RTs. If we were to use the speech produced by the children addressed in the CDS corpus to estimate AoFP, the difference in effect on AoFP between MWUs in CDS and MWUs in ADS might simply be due to the fact that both the dependent (AoFP) one of the independent variables (MWUs in CDS) have been estimated from related corpora. To avoid this issue, we estimate AoFP from an unrelated corpus.

**Figure 1:**
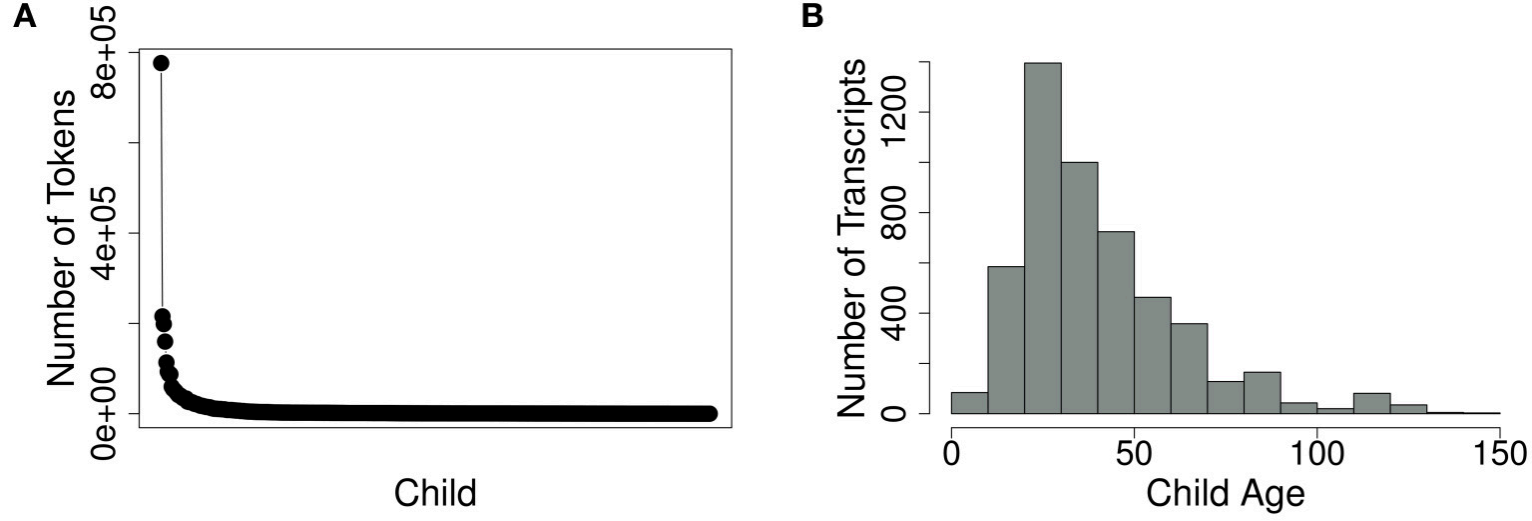
**Rank distribution of the number of tokens uttered by each child (A)** and distribution of transcripts by child age in months **(B)**.

The AoFP corpus is based on 44 American English corpora from the CHILDES database, which together contain 3,188,944 tokens produced by 463 children. The number of tokens contributed by individual children varies, with large longitudinal studies contributing a few thousand tokens for a single child each and some cross-sectional studies contributing only hundreds of words per child (cf. Figure 1A). The children in most transcripts are between 10 and 70 months old, with relatively fewer transcripts for children between 1 and 10 or 70 and 150 months (cf. Figure 1B).

In a balanced data set, utterances for each child would span the same age ranges, with the same number of words for each child—and consequently, the distributions in Figures 1A,B would be completely flat. We could then identify first usages in each child's data and take the mean, obtaining an average AoFP value for every target word. But because of the current uneven distributions, such a procedure would introduce noise into the AoFP estimates. Suppose, for example, that we have data from one child for ages 2 –5, and data from ten additional children for ages 5–6. Suppose, furthermore, that the younger child uses a particular word for the first time at age 3, while all the older children use it from the earliest recorded time on (age 5). In cases like these, it is plausible to assume that most of the older children would have been using the word in question since well before their data were collected. Thus, by including their first usages in the average AoFP, we would artificially inflate the estimate.

To avoid this issue, we treat a word as having been learned at the earliest developmental stage at which any child within the corpus produces it. In doing so, it is possible that we still include first usages which are also artificially inflated (because the child may have been using the word prior to the commencement of data collection), but at least we do not exacerbate the problem by averaging across AoFP values. In spite of these precautions, it is still possible that our AoFP estimates do not, after all, correspond very closely to the ages at which children learn words. To ensure methodological validity, we thus correlate AoFP with age of acquisition estimates collected via an elicited production task. The correlation is strongly positive (see Section 3.1.4 below), strengthening our confidence in the AoFP estimates.

*Developmental stage* is defined in terms of mean length of utterance (MLU)—the average child utterance length, in tokens, within a transcript. We induce MLU rather than AoFP estimate because children who are close in age may nevertheless be far apart in terms of language development. Being a more robust estimator, MLU controls for such developmental differences (Parker and Brorson, 2005). Since transcripts contain varying numbers of utterances, the average utterance length per transcript is biased with respect to transcript length. We rectify this issue by estimating MLU for each transcript via statistical bootstrapping, wherein the sampling distribution of the population is approximated by drawing random samples from the data (Davison and Hinkley, 1997). Each bootstrap is based on 1,000 random samples with replacement, with the sample size equal to the number of child utterances per transcript. We thus induce MLU rather than AoFP estimates but will, for simplicity, refer to a word's MLU value as its AoFP. To induce a value for a given word, we calculate the set of MLUs γ for all transcripts within which the word appears and assign it the smallest value in γ. We perform this procedure for all 29,055 word types identified via this method.

#### 3.1.3. Adult reaction times

The second dependent variable—RTs from a lexical decision task—measures word recognition in adults. Following the word recognition literature, we assume that words with fast RTs are easier to recognize than words with slow RTs. A negative correlation between e.g., ADS-#Freq and RTs would then indicate that words which are frequent in ADS tend to be quickly recognized; and a possible interpretation would be that frequency of exposure leads to fast word recognition in adults by strengthening the word's representation in long-term memory.

RTs are taken from the English Lexicon Project (Balota et al., 2007), which contains RTs from a lexical decision task for 40,481 mono- and multi-syllabic English words. Data were collected from participants recruited at six different U.S. universities (mean age ≈ 23 years)—meaning that just as AoFP, RTs were collected from native speakers of American English.

In the lexical decision task, subjects were presented with a string of letters corresponding to either an English word or a non-word, following which they were required to press a button if they thought the string was a word and another button if they thought the string did not correspond to a word. The time taken between stimulus presentation and button press was averaged across participants, resulting in a mean RT estimate for each word.

#### 3.1.4. Validity of AoFP and relationships among dependent variables

With the dependent variables in place, it is important to ensure methodological validity of the AoFP estimates. The advantage of using a collection of CHILDES corpora to estimate AoFP lies in the large number of words we can cover, but it is nevertheless desirable to compare AoFP to estimates elicited in controlled experiments. In addition, we ought to verify that AoFP and RTs are not too strongly correlated—to avoid potential difficulties in interpreting correlations with the independent variables, as well as to ensure that AoFP and RTs measure different underlying processes.

Our approach is methodologically related to work concerned with *age of acquisition*. Beginning with Carroll and White (1973), a large number of researchers have used adult estimates of when they learned to understand or use specific words to predict adult performance on various tasks (Barry et al., 2001; Bonin et al., 2004; Brysbaert and Cortese, 2010). However, this way of estimating age of acquisition may raise methodological concerns, as adult memory for childhood learning is very inaccurate (Baayen et al., 2016). To address this issue, Morrison et al. (1997) had children of varying ages perform a picture naming task. If a child is able to produce the correct noun (the picture name), he or she can be said to have learned the word. Presumably because of time constraints, Morrison et al. (1997) provide age of acquisition for a restricted set of 297 picturable nouns.

While the restricted focus makes their data less suitable for our analyses, Morrison et al. (1997)'s data are the only age of acquisition estimates for English that are directly derived from children. If our AoFP estimates are methodologically valid, we should expect their ordering to be strongly positively correlated with the order of Morrison et al. (1997)'s age of acquisition data. And indeed, for the 277 words shared between the two data sets, Spearman's *rho* is 0.61 (*p* ≤ 10^−20^), strengthening our confidence in the validity of AoFP. RTs from the English Lexicon Project correlate less strongly with both age of acquisition (ρ = 0.35, with *p* ≤ 10^−8^, for 284 shared words) as well as AoFP (ρ = 0.31, with *p* ≤ 10^−20^, for 10,883 shared words), suggesting that age of acquisition/AoFP and adult RTs measure different underlying processes.

#### 3.1.5. Statistical analysis

For the choice of correlation coefficient, we use a particular formulation of Kendall's coefficient, Kendall's τ*-b*, as it addresses potential pitfalls with the data. Consider Table 4 as a snapshot of the available data, where each row represents a target word. From left to right, each column contains: the target word, its frequency of occurrence in CDS (CDS-#Freq), the number of unique MWUs within CDS that contain it (CDS-#MWUs), and the word's age of first production (AoFP). Two of the correlations we wish to examine are (1) CDS-#Freq vs. AoFP and (2) CDS-#MWUs vs. AoFP. Being frequency counts, both CDS-#MWUs and CDS-#Freq are non-normally distributed. In addition, data points are often tied on these two variables—i.e., they have the same value for either one or both. For example, the last two rows in Table 4 are tied on CDS-#Freq, and the last three rows are tied on CDS-#MWUs.

**Table 4:** **Example data points**.

**Word**	**CDS-#Freq**	**CDS-#MWUs**	**AoFP**
Mummy	11,265	5,298	0.804
Said	4,894	2,357	1.111
Body	180	69	1.209
Learn	162	69	2.405
Covered	162	69	1.951

*Statistics are estimated from the CDS corpus*.

Kendall's τ*-b* addresses both issues. Unlike Pearson's *r*, which requires normality and is sensitive to outliers, τ*-b* makes no assumptions about the distribution of variables. And unlike Spearman's ρ, τ*-b* explicitly addresses tied data points (Agresti, 2010). Intuitively, given two different orderings of a set of data points, τ*-b* is a function of the number of data pairs which appear in the same order within both orderings, minus the number of pairs that appear in different orders. τ*-b* thus compares rankings of data points rather than real values. The approach taken, moreover, is maximally general, ensuring resistance to discrepancies in the data. This generality comes with a decrease in statistical power; but this is compensated for by the amount of data, as we work with close to 7,500 target words.

Moving toward a more concrete description of τ*-b*, let *X*, *Y* be the rankings of target words according to two different variables (e.g., CDS-#MWUs and AoFP). A pair of target words *t*_*i*_, *t*_*j*_ are then assigned ranks *x*_*i*_, *x*_*j*_ ∈ *X* and ranks *y*_*i*_, *y*_*j*_ ∈ *Y*. The two pairs of ranks are *concordant* if they appear in the same order, i.e., if either *x*_*i*_ < *x*_*j*_ ∧ *y*_*i*_ < *y*_*j*_ or *x*_*i*_ > *x*_*j*_ ∧ *y*_*i*_ > *y*_*j*_. If the two pairs are ordered differently, they are *discordant*. Given the number of concordant pairs *P* and the number of discordant pairs *Q*, the correlation coefficient of *X* and *Y* is calculated as follows:

$$\begin{array}{l} \tau -b_{XY}=\frac{P-Q}{\sqrt{\left(P+Q+X_{0}\right)\left(P+Q+Y_{0}\right)}} \end{array}$$

where *X*_0_ is the number of pairs tied only in *X* and *Y*_0_ is the number of pairs tied only in *Y* (pairs tied in both rankings are not considered).

In addition to such pairwise correlations, we calculate partial correlations—for example, we may want to correlate CDS-#MWUs and AoFP, controlling for CDS-#Freq. A partial correlation would then remove the variance shared between CDS-#Freq, CDS-#MWUs, and AoFP. Controlling for the ranking by a third variable (*F*), partial τ*-b* of the rankings *X* and *Y* is given by:

$$\begin{array}{l} \tau -b_{XY,F}=\frac{\tau -b_{XY}-\tau -b_{FX}\times \tau -b_{FY}}{\sqrt{\left(1-\tau -b_{FX}^{2}\right)\times \left(1-\tau -b_{FY}^{2}\right)}} \end{array}$$

Ninety-five percent confidence intervals for correlation coefficients are calculated via statistical bootstrapping (Davison and Hinkley, 1997), with each bootstrap based on 1,000 random samples with replacement, and a sample size equal to the number of data points. When comparing two correlation coefficients, we bootstrap 95% confidence intervals for the difference between coefficients (again based on 1,000 random samples with replacement). If zero is not contained within this interval, we can claim with 95% certainty that the two correlation coefficients differ from one another.

### 3.2. Results and discussion

**Figure 2:**
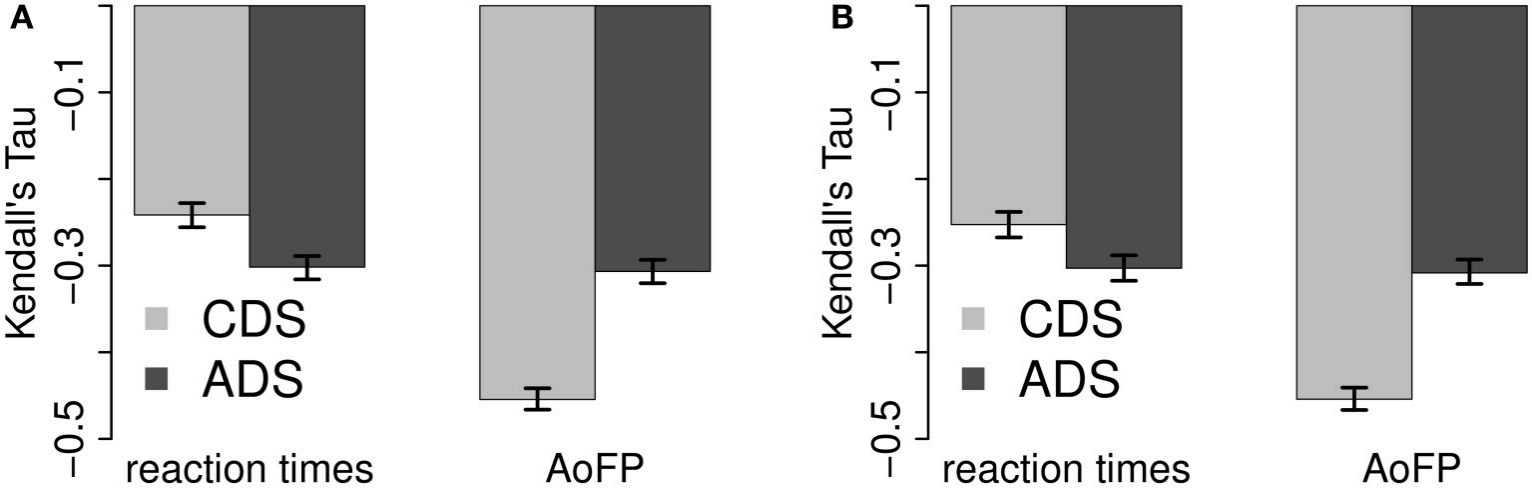
**Full pairwise correlations with ADS and CDS predictor variants. (A)** Correlations with #Freq. **(B)** Correlations with #MWUs.

**Figure 3:**
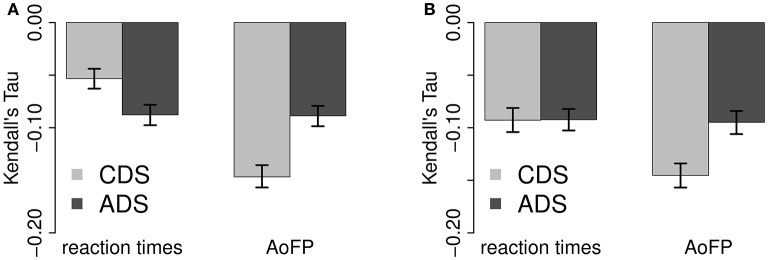
**Partial pairwise correlations with ADS and CDS predictor variants. (A)** Correlations with #Freq. **(B)** Correlations with #MWUs.

Correlations between response variables and predictors are summarized in Figures 2, 3 (see Appendix B in Supplementary Material for exact values). Figure 2 shows full pairwise correlations for #Freq (Figure 2A) and #MWUs (Figure 2B). #Freq and #MWUs are negatively correlated with RTs and AoFP: the more frequent a target word is and the more MWUs contain it, the earlier the target is produced by children, and the faster it is identified in a lexical decision task by adults. Furthermore, it does not matter whether we use #Freq or #MWUs: the overall picture is very similar, with AoFP being more strongly negatively correlated with CDS-derived predictors, while RTs are more strongly negatively correlated with ADS-derived predictors (95% confidence interval for the absolute difference between the full correlations of RTs with ADS-#Freq and CDS-#Freq: 0.05–0.07; RTs with ADS-#MWUs and CDS-#MWUs: 0.04–0.06; AoFP with CDS-#Freq and ADS-#Freq: 0.14–0.16; and AoFP with CDS-#MWUs and ADS-#MWUs: 0.13–0.16).

Figure 3 shows the corresponding partial correlations. Controlling for #MWUs (Figure 3A), RTs are still more strongly negatively correlated with ADS-#Freq, and AoFP is still more strongly negatively correlated with CDS-#Freq (95% CI for the absolute difference between the partial correlations with RTs: 0.02–0.05; and with AoFP: 0.05–0.07). Controlling for #Freq, (Figure 3B), CDS-#MWUs is still more strongly negatively correlated with AoFP (95% CI for the absolute difference: 0.04–0.06), while there is no significant difference between the correlations of RTs with CDS-#MWUs and with ADS-#MWUs (95% CI: 0.00–0.01).

We thus have reason to suspect that the frequency of words in ADS affects RTs more strongly than the frequency of words in CDS. Similarly, the frequency of words in CDS appears to have a stronger effect on AoFP than frequency in ADS. The results furthermore suggest that MWUs in CDS affect AoFP more strongly. We cannot, however, detect a difference between the independent effects of MWUs in ADS and MWUs in CDS on RTs. The general trend is, nevertheless, quite clear: the ADS predictor variants are more strongly correlated with RTs, while the CDS variants are more strongly correlated with AoFP.

In summary, both predictors are negatively correlated with RTs and AoFP—suggesting that frequency and MWUs facilitate both word learning in children and word recognition in adults. Furthermore, the ADS variants are generally more strongly correlated with RTs, and the CDS variants are generally more strongly correlated with AoFP—validating the assumption that ADS is the relevant linguistic input for adults, while CDS is the relevant source of input for children. For the remaining comparison, we choose to correlate ADS-#Freq as well as ADS-#MWUs with RTs, and CDS-#Freq as well as CDS-#MWUs with AoFP. That is, we consider (1) the effect of both predictors from ADS on RTs and (2) the effect of both predictors from CDS on AoFP.

## 4. Analysis III: comparison with a random baseline

We have established that #MWUs has a facilitatory effect on AoFP and RTs, and that this effect cannot be reduced to the frequency of target words. However, words which appear in a large number of MWUs could be likely to also appear in a large number of randomly selected word sequences. As a consequence, the effect of #MWUs on the two response variables could be due to collinearity with the number of random sequences within which each target word occurs. We use a random baseline to control for this possibility.

### 4.1. Method

Recall that the baseline model is a mirror version of the CBL, except insofar as it uses a random decision to group successive words into sequences, instead of the backward transitional probabilities used by the CBL. As a result, the MWUs discovered by the CBL are cohesive sequences of words, whereas the sequences extracted by the baseline lack this internal cohesion. In analogy to the #MWUs measure, we count the number of baseline-extracted word sequences within which each target word appears, and we denote this measure *#baseline*.

The target words and statistical analysis remain unchanged from the previous analysis. And as in analysis II, we compare correlation coefficients—namely, we compare the correlations of AoFP and RTs with #MWUs to the corresponding correlations with #baseline. If there is a unique facilitatory effect of #MWUs, the correlations with #MWUs should be stronger than the correlations with #baseline.

### 4.2. Results and discussion

**Figure 4:**
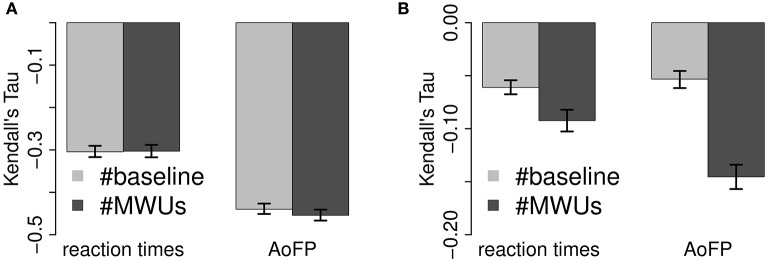
**Comparison of correlations with MWUs from the Chunk-Based Learner and word sequences from a model which randomly groups words into MWUs. (A)** Full correlations. **(B)** Partial correlations.

As explained in the foregoing analysis, we correlate ADS-#MWUs with RTs and CDS-#MWUs with AoFP. For the current analysis, this means that we compare (1) the correlation of ADS-#MWUs with RTs to the correlation of ADS-#baseline with RTs and (2) the correlation of CDS-#MWUs with AoFP to the correlation of CDS-#baseline with AoFP. These comparisons are summarized in Figure 4.

We cannot detect a statistically significant difference between the full correlations of #MWUs and #baseline with the two response variables (Figure 4A) (95% confidence interval for the absolute difference between the full correlations with RTs: 0.00–0.00; and with AoFP: 0.00–0.01). However, a significant difference emerges once we control for #Freq (Figure 4B): while both #MWUs and #baseline are negatively correlated with RTs and AoFP, the partial correlations with #MWUs are stronger (95% confidence interval for the absolute difference between the partial correlations with RTs: 0.02–0.04; and with AoFP: 0.08–0.10). Given that a difference emerges once frequency is controlled for, the absence of a difference between the full correlation coefficients is likely an artifact of frequency. In other words: compared to #baseline, #MWUs is in fact the stronger predictor. MWUs defined on BTPs between successive pairs of words are, therefore, likely to uniquely facilitate both child word learning and adult word recognition—above and beyond what could be explained by either frequency of exposure or a random baseline.

## 5. Analysis IV: comparing the effect of multi-word units on word learning to the effect on word recognition

The aim of this last analysis is to compare the effect of MWUs across word learning in children and word recognition in adults. That is, we attempt to ascertain which of the two areas is more strongly affected by MWUs. We have no reason to expect a stronger effect on either area. Given that children's first lexical representation are likely fossilized MWUs, it is possible that MWUs have a particularly strong effect on word learning and a comparatively weaker effect on adult lexical processing; but it is also possible that MWU representations become more entrenched over the course of development, resulting in an even stronger effect on adult word recognition.

### 5.1. Method

The target words and statistical method remain unchanged from the previous two analyses. Here, we compare the correlation with each predictor across the dependent variables. That is, we ask which of the two predictors has a stronger potential impact on AoFP, and which has a stronger potential impact on RTs.

### 5.2. Results and discussion

**Figure 5:**
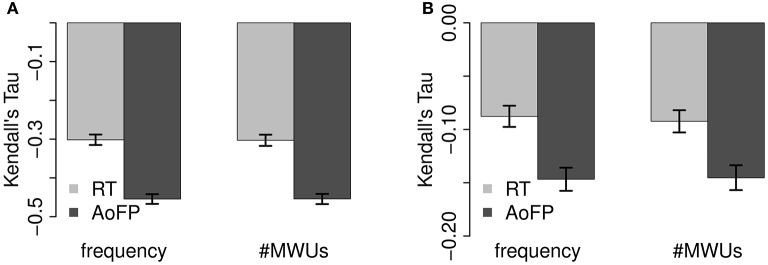
**Comparison of correlations with predictors across dependent variable. (A)** Full correlations. **(B)** Partial correlations.

Recall that we use the ADS predictor variants for correlations with RTs and the CDS variants for correlations with AoFP, i.e., we correlate ADS-#Freq and ADS-#MWUs with RTs, and CDS-#Freq and CDS-#MWUs with AoFP. This means that we compare (1) the correlation of ADS-#Freq with RTs to the correlation of CDS-#Freq with AoFP, and (2) the correlation of ADS-#MWUs with RTs to the correlation of CDS-#MWUs with AoFP. Figure 5 visualizes these comparisons.

The full correlations (Figure 5A) of CDS-#Freq and CDS-#MWUs with AoFP are more strongly negative than the correlations of the corresponding ADS predictor variants with RTs (95% CI for the absolute difference between ADS-#Freq vs. RTs and CDS-#Freq vs. AoFP: 0.13–0.17; ADS-#MWUs vs. RTs and CDS-#MWUs vs. AoFP: 0.13–0.17). This state of affairs remains unchanged when we control for the other predictor (Figure 5B) (95% CI for the absolute difference between ADS-#Freq vs. RTs and CDS-#Freq vs. AoFP: 0.04–0.07; ADS-#MWUs vs. RTs and CDS-#MWUs vs. AoFP: 0.04–0.07). Thus, even when factoring out MWUs, the effect of CDS-#Freq on AoFP is stronger than the effect of ADS-#Freq on RTs; and even when factoring out frequency, the effects of CDS-#MWUs on AoFP is stronger than the effect of ADS-#MWUs on RTs. This pattern suggests that frequency and MWUs have a stronger effect on child word learning and a relatively weaker effect on adult word recognition.

## 6. General discussion

### 6.1. General effect of multi-word units

The analyses reported above revealed a negative correlation between the two response variables and the number of MWUs within which words appear. That is, words which appear in relatively many of the MWUs discovered by the CBL tend to be first produced at comparatively early stages in development and tend to be identified relatively quickly by adult subjects in a lexical decision task. Importantly, the correlations surpass the effect of a random baseline and persist even when the frequency of target words is controlled for.

We also found a negative correlation between the two response variables and the frequency of individual words. This is not surprising: word frequency has been established as a predictor of word recognition (Balota et al., 2004), with more frequent words being recognized more quickly. In language acquisition, frequency effects are likewise well-attested—including a positive effect of frequency on the age at which children learn words (Ambridge et al., 2015). The effect of #MWUs, on the other hand, constitutes novel evidence for a beneficial impact of MWUs on both word learning and word recognition.

We began this paper by proposing the *MWU acquisition hypothesis*, according to which the formation of MWU representations precedes and then facilitates the formation of single-word representations. On the basis of this hypothesis, we expected that words contained in relatively many of the MWUs discovered by our model will be learned comparatively early in development. This prediction is borne out by the negative correlation of AoFP and the number of MWUs per target word. We also hypothesized that MWU representations facilitate adult word recognition (*MWU processing hypothesis*), leading us to expect that words contained in many model-derived MWUs will be quickly recognized by adults in a lexical decision task. The negative correlation of RTs and the number of MWUs per target word substantiates this prediction.

With the evidence in place, what is lacking is a compelling account of *how* MWUs affect learning and processing. While possible explanations are necessarily going to be exploratory, we nevertheless attempt to synthesize and build on insights from the literature.

### 6.2. Multi-word units in word learning

While we proposed the *MWU acquisition hypothesis* based on existing evidence, it is unclear exactly how MWUs exert their facilitatory effect on word learning. Learning to use words is a complex task – subsuming, among other things, the segmentation of phonological forms (Saffran et al., 1996), understanding the intentions of others (Baldwin, 1991; Carpenter et al., 1998), and integrating information across sensorimotor modalities (Lakoff, 1987; Barsalou, 1999). MWUs could potentially interact with several of these processes.

Word segmentation is, perhaps, the most probable candidate process. Consider Peters's (1983) proposal that early-acquired MWUs, being stored in long-term memory, are gradually segmented into smaller units—units which are themselves stored in memory, where they are again subject to segmentation. In this fashion, children could bootstrap small-grained linguistic units from an initial inventory of larger chunks. Later work concerned with children's early productions supports this view, showing that in spite of between-child differences in the degree of reliance on initial storage of unanalyzed patterns, all children may in fact rely on this strategy to some extent (Pine and Lieven, 1993). Evidence from perception studies, meanwhile, suggests that infants segment and store both actual and possible words—phonological forms which are word-like but do not correspond to words (Marchetto and Bonatti, 2013; Ngon et al., 2013). In light of the evidence from production, it is plausible that some of these early-segmented units contain several words—i.e., that children sometimes segment multi-word chunks before they begin to segment individual words from within those chunks. Thus, some early fossilized MWUs are likely to be (partially) undersegmented chunks (this could, for example, apply to the MWU *find-it*, used by Tomasello (1992)'s daughter to express desire for an absent object). In this scenario, the more initially undersegmented MWUs contain a given word, the earlier it is going to be segmented. We would then expect this early segmentation to translate into early induction of meaning, as children would have more time to establish the word's meaning, compared to late-segmented words.

The CBL, however, does not start from unanalyzed sequences. Instead, it operates on fully segmented words and builds those up into MWUs. In spite of this, the units it discovers still overlap with the chunks discovered by a simple segmentation algorithm. McCauley et al. (2015) compared the CBL to a segmentation method which initially stores whole utterances (represented as continuous streams of phonemes) as potential words and splits future utterances based on stored exemplars. Comparing the units discovered by the two methods, at identical points in time, shows that some MWUs are discovered by both algorithms. Thus, the MWUs discovered by the CBL correspond, to some extent, to chunks which could result from the initial storage of unanalyzed input^1^.

Of course, under-segmentation need not be the only way in which children form MWU representations. It is possible that the combination of smaller units proceeds side-by-side with segmentation, and that the two methods constitute complementary ways of discovering MWUs (McCauley et al., 2015). There may thus be additional processes involved in word learning, beyond segmentation, that interact with MWUs. Consider the process of establishing a word's meaning: following successful segmentation, to establish the meaning of an object name, children need to create a link between word form and object referent. Estes et al. (2007) have shown that children are capable of executing these two steps in sequence. In the first of two experimental phases, infants listened to a sequence of syllables containing an easily segmented phonological form. In a subsequent object-label-learning task, this phonological form was presented together with a set of novel forms. Infant subjects were able to map the phonological form from the previous phase to an object, but failed to do so with the novel forms. In principle, children are thus able to first segment a meaningless sequence of sound from an incoming speech stream; later, they are able to recognize the sequence within a new context and can map it to a referent.

Crucially, this series of steps involves the recognition of a stored word form before its meaning is established. And it is here that MWUs could again facilitate word learning. To see how, suppose that the subjects in Estes et al. (2007)'s experiment have segmented a phonological form, stored it in memory, and are about to retrieve it in order to map it to a referent. Suppose that this word form is part of one or more MWU representations. Children would then have access to fully-fledged MWU representations without having access to the meaning of each individual word. When encountered, some word forms could thus be more quickly retrieved from memory because they are part of one or more MWUs: if a word form, through MWUs, is linked to many other words, it should be more easily primed for retrieval from memory. And if it is easier to retrieve a word form from memory, we would expect fewer necessary exposures to word forms and their referents to establish a link between the two, compared to word forms which are part of relatively few MWUs.

We have thus identified two possible mechanisms, word segmentation and retrieval of word forms from memory, that are likely to be part of word learning; and we have spelled out ways in which MWUs could interact with these mechanisms. In both cases, MWUs are expected to have a beneficial impact: the more MWUs a given word is contained in, the easier it should be to segment the word from an unsegmented stream of speech, and the easier it should be to retrieve it from memory prior to establishing its referent.

### 6.3. Multi-word units in word recognition

While we referred to the literature on word recognition and contextual diversity as the foundation for the *MWU processing hypothesis*, it is not clear exactly how MWUs facilitate word recognition. One possibility is hinted at in the preceding section and has to do with the retrieval of word forms from memory.

Just like word learning, word recognition relies on a memory component: recognizing a string of letters as corresponding to a particular word form should certainly involve accessing a lexical representation. In fact, while prominent models of word recognition (Seidenberg and McClelland, 1989; Coltheart et al., 2001) differ in terms of implementation details, they agree on this core process. The beneficial impact of word frequency on RTs from lexical decision tasks can then be interpreted as as facilitatory effect of exposure on retrieval of words from memory. This basic mechanism is implemented in Seidenberg and McClelland (1989)'s connectionist model, which strengthens neuronal connections involved in processing specific words with every exposure; or in Coltheart et al. (2001)'s lexical model, which imposes frequency-based accessibility thresholds on word representations.

Revised or additional mechanisms are needed to accommodate findings pertaining to contextual diversity. Referring to the rational analysis of memory (Anderson and Milson, 1989; Anderson and Schooler, 1991), Adelman et al. (2006) suggest that word accessibility could be governed by likely need, arguing that the more contexts a word appears in, the higher the likelihood that the word will be needed in new contexts. Alternatively, a recent line of work suggests that language processing involves an expectation generation mechanism, which facilitates processing of highly predictable words and reduces memory load for such words (Altmann and Mirković, 2009; Elman, 2009). Based on this idea, Johns et al. (2014) suggest that highly contextually diverse words, being difficult to predict, are more reliant on strong memory representations. Thus, although the specifics are uncertain, these possible explanations have in common that memory takes center stage.

Note that these proposals carry over to an MWU-based view. Contextual diversity is generally quantified as the number of documents or paragraphs within which words appear. We have proposed that contextual diversity causes the internal linking-together of words into MWUs, and that this is what ultimately causes measurable effects such as a correlation with RTs. This explanatory shift is based on a number of studies showing that both children (Bannard and Matthews, 2008) and adults (Arnon and Snider, 2010; Arnon and Priva, 2014; Arnon et al., 2017) are likely to form MWU representations. In other words, if people link words to one another to form MWU representations, then MWUs —not paragraphs or documents—are the relevant cognitive units. Consequently, it should be the interaction between MWU and single word representations which facilitates word recognition, not the interaction between words and documents/paragraphs. The idea that MWU and single word representations interact, in various ways, during the processing of both MWUs and individual words is supported by previous work: Sprenger et al. (2006) showed that idiomatic phrases both prime and are primed by their constituent words, while Jacobs et al. (2016) found that equally frequent adjective-noun phrases are more easily recognized if they contain an easily recognizable noun.

Thus, if we accept the idea of a facilitatory interaction between word and MWU representations, Adelman et al. (2006)'s and Johns et al. (2014)'s suggested explanations for the beneficial effect of contextual diversity on word recognition carry over to our results with MWUs. Adapting Adelman et al. (2006)'s proposal to an MWU-based framework, it is also possible that the more MWUs a word appears in, the greater the likelihood that it will be needed in new contexts, and this is why such words have particularly accessible memory representation. And adapting Johns et al. (2014)'s suggestion, it could be that people utilize the mental links which form the basis for MWUs in order to predict upcoming words. This prediction process would then be less accurate for words contained in relatively many MWUs—hence necessitating stronger memory representations for such words.

To sum up, we have zeroed in on retrieval of words from memory as a core component of word recognition, and we have reviewed possible ways in which MWUs could interact with the retrieval process. Here, our core argument is that the more MWUs a given word is contained in, the faster it should be retrieved from memory, and the faster it should consequently be recognized.

### 6.4. Word learning vs. word recognition

We have attempted to sketch ways in which MWUs could benefit both word learning and word recognition, providing a possible explanation for (1) the negative correlation between #MWUs and AoFP and (2) the negative correlation between #MWUs and RTs. However, we have not yet considered the results of analysis IV, in which we compared the effects of #MWUs and #Freq across the two dependent variables. The results revealed (1) that CDS-#Freq was more strongly correlated with AoFP than ADS-#Freq with RTs and (2) that CDS-#MWUs was more strongly negatively correlated with AoFP than ADS-#MWUs with RTs. This suggests that frequency of occurrence and involvement in MWUs both affect word learning more strongly than word recognition and indicates a relatively stronger impact of language input during language acquisition, compared to the impact of input on adult processing. Generally speaking, this could be due to greater plasticity during language development and, as a consequence thereof, an increased sensitivity of children to various properties of the linguistic input.

The stronger potential impact of MWUs on word learning could also have to do with the range of cognitive processes which rely on MWUs. We have argued that both word learning and word recognition rely on retrieval of stored word forms from memory: during word learning, segmented word forms need to be retrieved in order to establish their meaning (e.g., to map them to a referent), while the retrieval of lexical representations from memory is so central to word recognition that the two are near-synonymous. In fact, we would argue that word learning subsumes word recognition, alongside many other sub-processes. If true, it is perhaps not unexpected that MWUs should have a stronger impact on word learning: if MWUs benefit the word recognition process, and if word learning subsumes word recognition in addition to other mechanisms which could likewise benefit from MWUs, then MWUs should have an overall stronger impact on word learning.

### 6.5. Limitations and open questions

Being correlational in nature, the results reported in this study are compatible with three interpretations: (1) direct causation, (2) spurious correlation, and (3) reversed causation. We have argued for possibility (1)—i.e., we have argued that involvement of words in MWUs directly causes them to be produced earlier and recognized more quickly than words which are not involved in (as many) MWUs.

While we have attempted to ground our interpretation of the observed correlations in previous results and observations, causality can only be established through future experiments with human subjects. For example, experiments along the lines of Johns et al. (2014), who demonstrated a causal link between contextual diversity and word recognition, could be fine-tuned to distinguish between general contextual diversity and MWUs. We would predict, based on the current findings, that MWUs are either the only or at least an additional contextual determinant of reaction times. Likewise, one could modify experiments along the lines of Estes et al. (2007), who demonstrated that infants can first store and then retrieve phonological forms for the purpose of linking them to a referent. In such a paradigm, learners could instead be exposed to novel word forms contained in MWUs that otherwise consist of known words, thereby eliminating the effect of segmentation. We would predict that such word forms will be rapidly linked to their object labels in a subsequent object-label-learning task.

## Author contributions

RG conceived of the original conceptual framework and design of this study and drafted the manuscript. GC was involved in critically revising and adding to the design, helped with the analysis, and made important additions to the the manuscript. SG and WD contributed critically to both the conception and design of this work and crucially revised the manuscript. All authors approved the final version for publication and agree to be accountable for all aspects of the work as well as to ensure that questions related to the accuracy or integrity of any part of the work are appropriately investigated and resolved.

## Funding

The present research was supported by a BOF/TOP grant (ID 29072) of the Research Council of the University of Antwerp.

### Conflict of interest statement

The authors declare that the research was conducted in the absence of any commercial or financial relationships that could be construed as a potential conflict of interest.

## Footnotes

^1^Note that CBL-derived MWUs would likely also overlap with chunks discovered via segmentation strategies that track transitional probabilities between syllables (Saffran et al., 1996). The CBL already tracks transitional probabilities between words, and most English words are monosyllabic. It follows that similar results would be obtained if we ran the CBL on (unsegmented) syllabified English utterances.
