# Supplementary material for: Facilitatory Effects of Multi-Word Units in Lexical Processing and Word Learning: A Computational Investigation
Source: Front Psychol. 2017 Apr 13;8:555. doi: 10.3389/fpsyg.2017.00555 (PMC5390038; doi:10.3389/fpsyg.2017.00555)
Supplement: Supplementary file 1 [file DataSheet1.pdf]

APPENDIX A: CHILDES CORPORA

1. CHILDES corpora used for CDS corpus:
   Belfast (Henry, 1995), Fletcher (Fletcher and Garman, 1988), Manchester (Theakston et al., 2001), Thomas (Lieven et al., 2009), Tommerdahl (Tommerdahl and Kilpatrick, 2013), Wells (Wells, 1981), Forrester (Forrester, 2002), Lara (Rowland and Fletcher, 2006)

2. CHILDES corpora used for AoFP corpus:
   Bates (Bates et al., 1991), Bernstein-Ratner (Ratner, 1986), Bliss (Bliss, 1988), Bloom 1970 (Bloom et al., 1974), Bloom 1973 (Bloom, 1976), Bohannon (Bohannon III and Marquis, 1977), Braunwald (Braunwald, 1971), Brent (Brent and Siskind, 2001), Brown (Brown, 1973), Carterette (Jones and Carterette, 1963), Clark (Clark, 1978), Cornell (no citation provided), Demetras (Demetras, 1986), Ervin-Tripp (no citation provided), Evans (no citation provided), Feldman (Feldman and Menn, 2003), Garvey (Garvey and Hogan, 1973), Gathercole (Gathercole, 1980), Gleason (Masur and Gleason, 1980), HSLLD (Beals, 1993), Hall (Hall et al., 1984), Higginson (Higginson, 1985), Kuczaj (Kuczaj, 1977), MacWhinney (MacWhinney, 1991), McCune (McCune, 1995), McMillan (no citation provided), Morisset (Morisset et al., 1995), Nelson (Nelson, 1989), NewEngland (Ninio et al., 1994), Peters/Wilson (Peters, 1987), Post (Demetras et al., 1986), Providence (Song et al., 2013), Rollins (Rollins, 2003), Sachs (Sachs, 1983), Snow (MacWhinney and Snow, 1990), Soderstrom (Soderstrom et al., 2008), Sprott (no citation provided), Suppes (Suppes, 1974), Tardif (no citation provided), Valian (Valian, 1991), Van Houten (Van Houten, 1986), Van Kleeck (no citation provided), Warren-Leubecker (Warren-Leubecker and Bohannon III, 1984), Weist (Weist and Zevenbergen, 2008)

APPENDIX B: CORRELATION COEFFICIENTS

		ADS-#Freq	CDS-#Freq	ADS-#MWUs	CDS-#MWUs	#baseline
full	RTs	-0.30 (-0.28, -0.31)	-0.24 (-0.22, -0.25)	-0.30 (-0.28, -0.31)	-0.25 (-0.23, -0.26)	-0.30 (-0.29, -0.31)
	AoFP	-0.30 (-0.29, -0.32)	-0.45 (-0.44, -0.46)	-0.30 (-0.29, -0.32)	-0.45 (-0.44, -0.46)	-0.45 (-0.44, -0.46)
partial	RTs	-0.08 (-0.07, -0.09)	-0.05 (-0.04, -0.06)	-0.09 (-0.08, -0.10)	-0.09 (-0.08, -0.10)	-0.06 (-0.05, -0.06)
	AoFP	-0.08 (-0.07, -0.09)	-0.14 (-0.13, -0.15)	-0.09 (-0.08, -0.10)	-0.14 (-0.13, -0.15)	-0.05 (-0.04, -0.06)

Table 5. Full and partial correlation coefficients with 95 % confidence intervals (in parentheses) for all correlations reported in analyses II – IV.
